# The Relationship between Internet Use and Health among Older Adults in China: The Mediating Role of Social Capital

**DOI:** 10.3390/healthcare9050559

**Published:** 2021-05-10

**Authors:** Yumei Zhu, Yifan Zhou, Cuihong Long, Chengzhi Yi

**Affiliations:** 1 School of Finance and Public Administration, Shanghai Lixin University of Accounting and Finance, Shanghai 201620, China; zhuyumei0123@163.com; 2 School of Political Science and Public Administration, East China University of Political Science and Law, Shanghai 201620, China; milklikexi@126.com; 3 School of Economics, East China Normal University, Shanghai 200062, China; chlong@jjx.ecnu.edu.cn; 4 School of International and Public Affairs, China Institute for Urban Governance, Shanghai Jiaotong University, Shanghai 200030, China

**Keywords:** internet use, physical health, mental health, social capital, older adults

## Abstract

A growing academic attention has been paid to the health effects of Internet use among older adults. However, the relationship between Internet use and health among older adults in China remains to be studied further. On the one hand, existing research is still controversial on this issue. On the other hand, the underlying mechanism of how Internet use affects the health of older adults has not been fully explored. This article examined the relationship between Internet use and health among older adults with the mediating role of social capital in China based on the 2018 wave of China Health and Retirement Longitudinal Study (CHARLS). This study reveals that Internet use has a positive association with the health of older adults, and the positive effects of internet use among older adults are heterogeneous in age and residential location. In addition, this study also demonstrates that social capital plays a partial mediating role between Internet use and physical health among older adults. It is important for the government to take effective measures to expand Internet use and enhance social capital among older adults.

## 1. Introduction

In recent years, with the acceleration of the “silver hair wave”, China has entered an aging society stage at a rapid rate and has become the country with the largest older adult population in the world [1]. National Bureau of Statistics data shows that the number of older adults aged 60 and above reached 253.88 million (approximately 254 million) in China by the end of 2019, which represents 18.1% of the total population [2]. Health status is closely related to age [3], and aging is considered to pose a serious health challenge [4]. In the context of accelerating aging and expanding size of the older adult population, the concern about the health of older adults has gradually become a prominent topic and has received widespread attention [5]. Specifically, the health status of Chinese older adults is not optimistic at present, as data released by the National Health Commission show that over 180 million people aged 60 and above in China are suffering from chronic diseases, and 75% of them suffer from chronic diseases co-morbidity [6]. The health status of older adults not only affects their own life, but also increases the pressure on the medical system and the society. Therefore, improving the health status of older adults is of great importance.

While the older adult population in China is constantly increasing, the widely application of the Internet has brought a new factor that could influence their health. In recent years, China’s Internet information technology is developing rapidly and the use of the Internet is also increasing and expanding continuously, which has brought profound changes and impacts on people’s way of life. Thus the lives of older adults are also greatly affected by the Internet. According to national statistics, the number of Chinese netizens is 904 million by the end of March 2020, and 6.7% of them are aged 60 and above [7]. The Internet continuously penetrates into the life of older adults all over the world and increasingly becomes a part of their life, which may have a health effect [8,9,10,11]. The use of the Internet can reduce the time and opportunities for people to communicate face to face [12], bringing fun and convenience to life [13], whilst the Internet addiction may also bring harm to user’s body and mind [14]. This shows the Internet use might have an impact on health among older adults.

Although existing studies have analyzed the health effects of Internet use [15,16,17,18,19,20,21], there haven’t been enough discussions focusing on the relationship between Internet use and health among older adults in China, and the conclusions of existing studies are still controversial [17,18]. If Internet use has an impact on the health of older adults, how does this impact happen? There is still a lack of satisfactory answers to this question. Internet use may not only have a direct impact on the health of older adults, but also have an indirect impact on the health of older adults through the mediating variable of social capital. As for the indirect impact of Internet use on the health of older adults, social capital may be a very important intermediary factor. The correlation between social capital and individual health has been analyzed by existing studies [22,23,24,25], and the use of the Internet may have an influence on the accumulation of social capital [26]. However, whether social capital plays a mediating role between Internet use and the health of older adults, and what kind of mediating role it plays remain to be further tested. In order to narrow the gap in existing research, we will use a large national sample to examine the relationship between Internet use and the health of older adults with the mediating effect of social capital in China.

The rest of this paper is arranged as follows: the second part introduces the materials and methods. The empirical results are analyzed in the third part. The fourth part is the discussion and the fifth part draws the research conclusions.

## 2. Materials and Methods

### 2.1. Literature Review

At present, the Internet is widely used in society globally and has become a way of life for more and more people, and the health effects of Internet use have also received increasing attention from researchers [17,18,19,20,21,27,28,29]. Some researchers have confirmed a positive correlation between Internet use and health among older adults. Xavier et al. found that English older adults who consistently used the Internet could be more likely to adopt cancer preventive behaviors [8]. The study of Long et al. revealed that Internet use had a positive contribution to increasing the psychological well-being of older adults in both rural and urban areas [16]. Research conducted by Ahn et al. suggested that Internet games indirectly affected the mental health of older adults in Korea, and this influence worked through the perception of self-control [9]. Moreover, Liu et al.’s study revealed that Internet use could reduce respondents’ depression via social participation [15]. Similarly, Cotton et al. also found that Internet use had a positive contribution to the mental well-being of the retired older adults in the United States [10]. In addition, the study of Song et al. suggested that the use of Internet could reduce individual loneliness significantly among older adults in China [11]. The research of Zhao et al. using data obtained from Chinese General Social Survey (CGSS), found that Internet use could promote the health of older adults in China, and the effects were mainly achieved by increasing the learning frequency of older adults [17]. However, some studies have different opinions on this issue. For example, Wang demonstrated that Internet use was helpful to enhance the psychological well-being of older adults in urban China, but this did not apply to those in rural China [18]. The study of Lu and Wang found that the overuse of the Internet was not good for health [21]. Moreover, the study of Yao and Zhong found that excessive and unhealthy use of the Internet could increase the feeling of loneliness [28]. In addition, Sanders et al. suggested that the relationship between Internet use and depression was not significant [29].

The abovementioned literature analyses show that researchers still hold different opinions on the relationship between Internet use and individual health, but most researchers tend to believe that Internet use could have an impact on individual health. If there is indeed a significant correlation between Internet use and individual health, what is the mechanism behind it? Internet use may not only be directly related to individual health, but also be indirectly related to individual health through intermediary variables, among which social capital could be a potential intermediary variable playing a role between Internet use and individual health. As an important concept in social sciences, social capital is used by researchers to refer to specific social resources [30], or a collection of social networks, norms, and trust [31]. Although different researchers have different definitions of social capital, they generally regard social capital as a multi-dimensional concept, and consider social connections, relationship or interaction as the core content of individual-level social capital [25,32]. On the one hand, Internet use is considered to be significantly related to social capital, but existing research on the direction of the relationship between Internet use and social capital is still controversial. A study suggests that the application of electronic technology could undermine the basis of social engagement [31]. Some researchers argue that Internet use may lead to a decline in social capital, as reflected by the decrease of social connections or networks [12,33].However, other researchers believe that there is a positive and significant relationship between Internet use and social capital [27,34,35].For example, a study finds that the use of Internet is conducive to enhancing communication capabilities due to its technical advantages and thus helps promote the accumulation of social capital [34]. On the other hand, social capital is considered beneficial to individual health [36]. A research conducted by Coll-Planas et al. revealed that social capital could be helpful in alleviating loneliness among older adults [37]. Therefore, social capital may play an intermediary role between Internet use and individual health. However, this intermediary role is still under explored.

Overall, a growing academic attention has been paid to the health effects of Internet use among older adults. However, the relationship between Internet use and health among older adults in China remains to be studied further. On the one hand, existing research is still controversial on this issue. On the other hand, the importance of social capital as a potential intermediary variable is still ignored. To fill this gap, we used the data that was obtained from the 2018 wave of CHARLS to explore the relationship between Internet use and health among older adults with the mediating role of social capital in China.

### 2.2. Data, Variables and Methods

#### 2.2.1. Data Source

The study used the data obtained from the 2018 wave of CHARLS. The survey is dedicated to providing high quality micro-data to analyze issues related to the aging population in China. Besides, the 2018 wave of CHARLS data used in this study is the latest wave of data available at present, which covers 19,816 respondents aged 45 and above in 450 communities or villages in China. Considering the needs and goals of research, we removed respondents under 60 as well as those observations that have missing values for the variable we are interested in, then a total of 6323 observations were included.

#### 2.2.2. Variable Design

##### Dependent Variables

In this study, the measurement of older adults’ health includes two parts: physical health and mental health. The Activity of Daily Living Scale (ADL) developed by Lawton and Brody in 1969, was employed to measure the physical health [38]. In CHARLS, older adults were asked whether they had difficulties in dressing, bathing, eating, getting into or out of bed, using the toilet, controlling urination and defecation. Based on the four responses to these questions, we recorded them from 1 for “I don’t have any difficulty” to 4 for “I cannot do it”. Then, we summed up the responses to obtain an ADL score, which reflects the older adults’ ability of daily living. In this study, the ADL score ranges from 6 to 24, and higher ADL score means worse physical health.

Furthermore, the Center of Epidemiological Survey-Depression Scale (CES-D) was adopted to measure the mental health, which was often used to examine the depressive symptoms in the general population [39]. In CHARLS, respondents were asked to answer ten questions involving their feelings and behaviors, and the potential responses to these ten questions include never, rarely, occasionally, mostly and all of the time. Among these ten questions, two questions are positively oriented and eight questions are negatively oriented, and this study used reverse scoring for positively oriented questions. Besides, we sum up the responses to obtain the CES-D score, which reflects older adults’ mental state. In this study, the CES-D score ranges from 10 to 40, the higher the CES-D score, the worse the mental state. 

##### Independent Variables

In this paper, Internet use is selected as the independent variable. Moreover, whether the Internet is used or not is adopted to measure the Internet use. In CHARLS, respondents were asked to answer the question “whether they used the Internet during the past month?” and we dichotomized their answers into “yes” = 1 and “no” = 0. Although whether to use the Internet is only a simple dimension for measuring Internet use, for the proportion of Internet use among older adults in China is very low, it is of great significance to analyze whether to use the Internet as the main independent variable. The latest data reveal that the number of Internet users in China has reached 989 million, 70.4% of the total population [40]. Our data analysis found that 93.3% of Chinese older adults do not use the Internet, and only 6.67% of older adults use the Internet. This shows that Internet usage rate of older adults in China is still very low, and it is still necessary to analyze the relationship between whether to use the Internet and the health of older adults in China.

##### Mediating Variables

Previous studies suggest that Internet use has a significant correlation with social capital [22], and social capital might have a contribution to health [22,36]. Based on this evidence, we hypothesize that Internet use may affect health through social capital. To test this hypothesis, we adopt social capital as an intermediary variable. Social capital is multi-dimensional and social network is an important dimension to measure social capital [23,31]. Research has pointed out that the use of a single dimension to measure social capital is widely applied in existing research [41], so this article mainly measures social capital from the perspective of social network. In CHARLS, respondents were asked to answer “Have you interacted with friends in the last month?” and for older adults who have contacted friends, they would be asked the further question “How often have you interacted with your friends in the past month?” We recorded the responses to 0 for “not interacted with friends”, 1 for “not regularly”, 2 for “almost every week”, and 3 for “almost daily”.

##### Control Variables

The previous studies suggested that individual characteristics, economic status and health-related behaviors significantly affect the health of older adults [42,43]. To obtain the effect of Internet use on health among older adults, some control variables were used in this study including gender, age, education level, political status, marital status, religious belief, household living expenditure and residential location. Besides, smoking status, drinking status and health insurance were also used in this study. In addition, this study also performed the multicollinearity tests, and the results show that the variance expansion factor (VIF) values of independent variables was much lower than 10, therefore we do not think that serious multicollinearity exists in the regression model.

The descriptive statistics of variables are shown in Table 1. The proportion of the older adults who use the Internet is 6.67%, which is a very low number compared to other groups. The mean of ADL score and CES-D score of the respondents is 5.601 and 18.776, respectively. And 56.16% of older adults in this study are aged 60–69, 60% of whom are married. Moreover, 74.02% of the respondents’ education level is primary school and below, 72.94% of the respondents are living in the rural areas, and the mean value of household living expenditure is RMB 14,701.45. In addition, 31.36% of the respondents have drunk in the past year, and 27.3% of them smoked.

#### 2.2.3. Methods

Given that the ADL score and CES-D score used in this research are continuous variables, the study uses the Ordinary Least Sqaure (OLS) model to examine the relationship between internet use and the health of older adults. The following econometric model is established in this paper:

$${\mathrm{Health}}_{i}=\alpha +{\beta \mathrm{Internet}}_{i}+{\gamma X}_{i}+\varepsilon_{i}$$

where i denotes the individual, ${\mathrm{Health}}_{i}$ indicates the health condition for the older adults, α is the intercept term, β and γ represents the regression coefficient for the corresponding variable, and $\varepsilon_{i}$ represents the error term.

## 3. Results

### 3.1. Baseline Regression Results Analysis

Table 2 displays the results of baseline regression estimation. As the estimation results show, the ADL score has a significantly negative relationship with Internet use, which confirms that older adults using the Internet have a lower ADL score and a better physical health state. Moreover, age, gender, education level, marital status, household living expenditure, smoking status, drinking status, and residential location have a significant association with the ADL score of older adults. Specifically, age and household living expenditure have a significantly positive correlation with the ADL score, indicating that the older people become and the more they spend on consumption, the less they are able to take care of themselves. In addition, political status, religious belief and health insurance have no significant association with the ADL score of older adults.

The regression results of Model 2-2 reveal that the CES-D score has a significantly negative association with Internet use. This shows that the older adults who use the Internet may have lower CES-D score, suggesting a better mental health condition. Moreover, it also finds that being older, male, having the education level of junior and senior high school, being the Chinese Communist party member, having religious belief, drinking, living in the urban area, and covered with health insurance are significantly related to lower CES-D score. Furthermore, receiving higher education and household living expenditure has no significant effect on the CES-D score.

### 3.2. Regression Results in Different Subgroups

In the above section, all respondents were treated as a homogeneous group and the average effect of Internet use on the health of older adults was analyzed. However, there might be a certain degree of heterogeneity in the use of the Internet among the older adults with different individual characteristics. Therefore, we also explored whether the association between Internet use and health differs by gender, age and residential location. The regression results by gender are shown in Table 3. 

As the estimation results of model 3-1 reveal, the correlation between Internet use and the ADL score of older adults for both female and male is significantly negative. The estimation results of model 3-2 show that the association between Internet use and the CES-D score of older adults for both female and male is also significantly negative.

Table 4 displays the regression results grouped by residential location. The estimation result of Model 4-1 shows that the correlation between Internet use and the ADL score is significantly negative for both urban and rural older adults. The estimation result of Model 4-2 reveals that the correlation between Internet use and the CES-D score is significantly negative in rural areas. But for the correlation between Internet use and the CES-D score in urban areas, the coefficient is not statistically significant and substantively less relevant. It means that Internet use is more helpful to the mental health of older adults living in rural areas. This is consistent with existing research findings [44]. Compared with the urban older adults, the rural older adults have relatively fewer medical and health resources. Health knowledge obtained from the Internet can help to make up for the shortage of rural medical and health resources.

Table 5 illustrates the regression results grouped by age. Generally speaking, there are differences in Internet use among older adults of different age groups, the proportion of Internet use among the younger groups are higher than older people. This study divides the older adults into two groups: aged 60–69 and aged 70 and above. Regression results of Model 5-1 reveal that Internet use has a significant correlation with the physical health of older adults aged 60–69 and aged 70 and above. Regression results of Model 5-2 show that Internet use also has a significant association with the mental health of older adults aged 60–69, but the correlation is not significant among older adults over the age of 70, which indicates that the influence of Internet use on the mental health of older adults in different age groups is heterogeneous.

### 3.3. The Treatment of Endogenity: Instrumental Variable Estimation

The Internet can help the older adults obtain information more conveniently and keep in touch with friends, which is helpful to improve their health, the older adults who are physically and mentally healthier have fewer difficulties in using the Internet and are more likely to master Internet use skills, which helps to promote their using of the Internet more frequently. In order to overcome the endogeneity bias caused by mutual causality, this study refers to the research by Yang and He [44], and selects the mean Internet use of the community where the respondents live as an instrumental variable for the Internet use of the respondents for the following reasons. Firstly, Internet communication is expansive. The more older adults residents in the community use the Internet, the more likely it will encourage other older adults to use the Internet, meanwhile the use of the Internet could have an impact on self-assessed health. In other words, the more frequently the older adults use the Internet on average in the community, the more likely they are to use the Internet which will influence self-assessed health through Internet use. Secondly, the behavior of residents living in the same community has a wide range of social interaction effects [45], which could also explain why the increasing average Internet use of older adults in a community would help to promote the Internet use of individuals in the community. Thirdly, generally speaking, the average Internet use of older adults living in the same community would not have a direct effect on their health. 

The estimation results of the instrumental variable model are reported in Table 6. In the first stage of regression, the F statistic is much larger than the empirical standard value of 10 [46], so it is believed that there is no problem of weak instrumental variables. Moreover, the results of the Durbin-Wu-Hausman test reveal that the variable of Internet use is endogenous. If the explanatory variable is an endogenous variable, the results obtained by using the instrumental variable estimation are more reliable. The estimation results of Model 6-1 show that after using the instrumental variable for estimation, there is still a significantly negative correlation between Internet use and the ADL Score. The estimation results of Model 6-2 also display that the correlation between Internet use and the CES-D Score remains significantly negative after introducing the instrumental variable. As a result, the relationship between Internet use and the health of older adults has been further verified.

### 3.4. Mechanism Analysis

This part aims to analyse the mechanism of Internet use affecting the health of older adults with the mediating role of social capital. Based on previous studies, social capital was adopted as a mediator variable, and the stepwise regression method proposed by Baron and Kenny [47] was used to analyze this relationship. Table 7 reports the results. Model 7-1 explores the association between the Internet use and the ADL score of older adults. From the results shown in the table, we can see that Internet use has a significantly negative association with the ADL score of older adults. Model 7-2 examines the association between Internet use and social capital, and the results reveal that Internet use has a positive association with the social capital of older adults. Based on the Model 7-1, Model 7-3 adds the mediator variable of social capital, and the results show that the correlation of Internet use with the ADL score of older adults is still significant while the beneficial association between Internet use and physical health of older adults has been weakened. Overall, the results suggest that the association between Internet use and the ADL score of older adults is partially mediated by social capital, which has provided a strong support for this research topic.

Furthermore, Model 7-4, Model 7-5, and Model 7-6 examine the mediating role of social capital between Internet use and the CES-D score of older adults. Model 7-5 reveals that the Internet use has a positive association with the social capital of older adults. However, Model 7-6 reveals that social capital has no significant association with the CES-D score of older adults, indicating that social capital does not play a mediating role between Internet use and mental health of older adults.

## 4. Discussion

In this study, we explored the association of Internet use with physical and mental health among older adults in China. Moreover, this paper also examined the different association between Internet use and the health of older adults by gender, age and residential location. Furthermore, we explored the mechanism of Internet use affecting the health of older adults through mediating analysis. The results reveal that the Internet use has a significantly negative association with the ADL score and the CES-D score of older adults, which indicates that Internet use contributes to enhancing the physical and mental health of older adults. This may be partly because that the information and resources on the Internet are more abundant than those on traditional media [48], which helps the older adults enrich their lives and maintain regular contact with friends, which is good for alleviating loneliness among older adults. Moreover, the Internet is also a good way of improving or monitoring health among older adults. Specifically, with the advancement of Internet technology, the older adults’ health may also benefit from the rapid development of wireless telemedicine and e-health services through Internet use. For example, many hospitals began to provide online healthcare or telemedicine services during the epidemic of COVID-19 [49], so that the older adults can obtain the health services conveniently through the Internet, especially for those with walking difficulties. And for the elderly patients who need to take medication regularly, they could be prescribed conveniently through telemedicine. In general, the Internet use could contribute to the health of older adults in many ways.

The regression results by age reveal that Internet use is more beneficial to the mental health of older adults under the age of 70. It can be implied that Internet use has a stronger association with the mental health of the younger older adults, which is consistent with the finding of Yang and He [44]. This may be partly because that the younger older adults have less difficulty using the Internet, so they can obtain various types of information and keep more contact with friends by using the Internet, which stands to reason that the social interaction as an element of social capital plays a certain role. However, there are more obstacles in the use of the Internet for the older adults over 70 years old, so their enthusiasm for using the Internet is not high, and the beneficial help of Internet use to their health is relatively limited. Compared with the younger older adults, those aged 70 and above have lower willingness and ability to learn how to use the Internet. It can be inferred from the data of age and residential location that the younger age and more connected residence signify a more extensive social network of older adults, and those who have more social capital are healthier.

The regression results by residential location reveal that the correlation of Internet use with the mental health of older adults is heterogeneous. Compared with the respondents living in urban areas, the mental health of older adults living in rural areas benefits more from Internet use, and this finding is consistent with the study of Yang & He [44]. It may be because the older adults living in rural areas have relatively fewer medical and health resources compared with their counterparts living in urban areas, and health knowledge obtained from the Internet can help to make up for the shortage of rural medical and health resources.

The conclusions of this research have several important policy implications. Firstly, it is quite necessary to improve the expanding use of the Internet among older adults. As is mentioned above, social support may affect the health of older adults to a certain extent. However, with the widespread application of information technology in daily life, the insufficient use of the Internet has brought a lot of inconvenience to older people’s lives, even causing a decline in the quality of life. For example, it is difficult for some older adults to enjoy the services such as online shopping and online car-hailing due to the fact that they don’t know how to use the internet, making them less socially connected and trusting on the Internet. During the COVID-19 epidemic in China, “the health code” has been widely used for identity verification in public places. Nevertheless, some older adults cannot show their health code because they do not know how to use the Internet, making it difficult to be authenticated in public places and use corresponding public services. In brief, those who cannot make good use of the Internet seem to have less social connections and social support, which furthermore affects the health of older adults to some extent. Consequently, the government needs to strengthen the network infrastructure and increase the opportunities for older adults to access the Internet. Secondly, the significant differences between the older adults in urban and rural areas urge that efforts should be made to increase the Internet popularity rate in rural areas, and narrow the gap between urban and rural Internet penetration. Also, it is necessary to provide more material assistance for the older adults in rural areas to enhance their social support and participation will. Thirdly, education and training on Internet use should be promoted so as to help older adults master the methods of Internet use and improve their skills, which is beneficial to realizing the sharing of health resources online, providing a rapid access to health information and then improving their health quality. Moreover, considering the heterogeneity of the impact of Internet use on the health among older adults, older people should be treated differently in the process of promoting Internet use among them, enhancing their sense of trust and belonging to the positive effects of the Internet use on health. In general, it is necessary to consider the different needs of older adults, and suitable Internet applications should be launched to provide better online experience for the older adults, so that they can enjoy a more convenient life brought by the Internet. In addition, it is also necessary to promote the development of telemedicine services as well as the improvement of e-health literacy among older adults, so that they can better enjoy the convenience brought by Internet in health services and treatment of diseases.

This study has several strengths. Firstly, this paper analyzed the influence mechanism of Internet use on the health of older adults by focusing on the mediating role of social capital. Not only does it complement the previous research and broaden the academic horizons, but also provides empirical support for improving the health status of older adults. Secondly, we use the latest wave of CHARLS data in this study, which can better reflect the new state of the Internet use of older adults and its association with the health of older people. Thirdly, this study suggests that building social capital is beneficial to improve health status of older adults. It is important for Internet service providers to speed up the establishment of a more open and standardized system and encourage the participation of Internet use among older adults.

Several limitations should be noted. Firstly, due to data limitation, the causal relationship between Internet use and the health of older adults still has to be explored and explained in future research. Secondly, the measure of respondents’ health status were based on self-reports, which may be affected by their personal feelings at that time, thus the bias may influence the results. Thirdly, given that this study only focused on the association between Internet use and the health of older adults, further researches of different contents of Internet use on health among older adults are needed.

## 5. Conclusions

To sum up, this study aims to probe into the mechanism of how Internet use affects the health of older adults under the mediating role of social capital, which reveals that Internet use has a positive association with the health of older adults mediated by social capital. To be more specific, the Internet use has a positive association with the social capital of older adults, and the correlation of Internet use with the ADL score of older adults is still significant when adding the mediator variable of social capital. Results show that the health of older adults is critically linked to the Internet, social support, age and residential location. These conclusions remain robust as the endogenous problem was handled by instrumental variable estimation. Moreover, the positive effect of Internet use among older adults is heterogeneous by age and residential location. The older adults at younger age or in rural areas show a healthier level than those of older age or in urban areas. In addition, this study also demonstrates that social capital plays a partial mediating role between Internet use and physical health among older adults. This suggests that the government should take effective measures to encourage and enhance the use of the Internet among older adults and expand the social network of older adults to improve their social capital in terms of Internet use.

## Figures and Tables

**Table 1:** Descriptive statistics of variables.

Variable	Description of Variables	*N* (%)	Mean	Standard Deviation
		(*N* = 6323)		
Age	60–69	3551 (56.16)	69.877	7.459
	≥70	2772 (43.84)		
Gender	Female = 0	3117 (49.3)	‒	‒
	male = 1	3206 (50.7)		
Education level	Primary school and below = 1	4680 (74.02)	‒	‒
	Junior high school = 2	997 (15.77)		
	senior high school = 3	543 (8.59)		
	higher education = 4	103 (1.63)		
Marital status	Single, divorced or widowed = 0	2120 (33.53)	‒	‒
	married = 1	4203 (66.47)		
Political status	Other = 0	5531 (87.47)	‒	‒
	the Chinese Communist party member = 1	792 (12.53)		
Religious belief	No = 0	5604 (88.63)	‒	‒
	Yes = 1	719 (11.37)		
Residential location	Living in rural areas = 0	4612 (72.94)	‒	‒
	Living in urban areas = 1	1711 (27.06)		
Household Living Expenditure	Continuous variable	*N* = 6323	14,701.45	47,672.72
Smoking status	No = 0	4597 (72.7)	‒	‒
	Yes = 1	1726 (27.3)		
Drinking status	No = 0	4340 (68.64)	‒	‒
	Yes = 1	1983 (31.36)		
Health insurance	Without health insurance = 0	197 (3.12)	‒	‒
	Covered health insurance = 1	6126 (96.88)		
Internet use	No = 0	5901 (93.33)	‒	‒
	Yes = 1	422 (6.67)		
social capital	Not interacted with friends = 0	4301 (68.02)	‒	‒
	Not regularly = 1	925 (14.63)		
	Almost every week = 2	410 (6.48)		
	Almost daily = 3	687 (10.87)		
ADL score	Continuous variable	*N* = 6323	5.601	3.548
CES-D score	Continuous variable	*N* = 6323	18.776	8.764

Source: CHARLS2018.

**Table 2:** Baseline Regression Results.

Variable	Model 2-1	Model 2-2
	ADL Score (Physical Health)	CES-D Score (Mental Health)
Internet use	−0.690 ***	−1.891 ***
	(0.182)	(0.466)
Age	0.098 ***	−0.199 ***
	(0.006)	(0.016)
gender	−0.795 ***	−1.741 ***
	(0.106)	(0.271)
Education level ^a^		
Junior high school	−0.513 ***	−1.225 ***
	(0.123)	(0.314)
Senior high school	−0.637 ***	−2.053 ***
	(0.165)	(0.422)
Higher education	−0.785 **	−1.129
	(0.347)	(0.888)
Marital status	−0.532 ***	0.681 ***
	(0.099)	(0.254)
Political status	−0.123	−0.603 *
	(0.136)	(0.349)
Religious belief	−0.200	−0.819 **
	(0.133)	(0.340)
Household living expenditure (ln)	0.111 ***	0.007
	(0.019)	(0.049)
Smoking status	−0.364 ***	0.532 *
	(0.107)	(0.274)
Drinking status	−0.675 ***	−0.729 ***
	(0.098)	(0.251)
Residential location	−0.358 ***	−1.781 ***
	(0.103)	(0.264)
Health insurance	−0.361	−1.481 **
	(0.241)	(0.618)
Constant	−0.366	35.772 ***
	(0.547)	(1.400)
Observations	6323	6323
*R*^2^R-squared	0.137	0.071
Adjusted *R*^2^	0.135	0.069

Note: Standard errors in brackets, * *p* < 0.1, ** *p* < 0.05, *** *p* < 0.01; ^a^ means primary school and below.

**Table 3:** Regression Results by Gender.

Variable	Model 3-1		Model 3-2	
	ADL (Physical Health)		CES-D (Mental Health)	
	Female	Male	Female	Male
Internet use	−0.586 **	−0.797 ***	−1.822 **	−1.842 ***
	(0.281)	(0.243)	(0.827)	(0.532)
Age	0.107 ***	0.0875 ***	−0.270 ***	−0.128 ***
	(0.008)	(0.009)	(0.024)	(0.020)
Education level ^a^				
Junior high school	−0.467 **	−0.531 ***	−1.839 ***	−0.786 **
	(0.196)	(0.160)	(0.578)	(0.351)
Senior high school	−0.416	−0.745 ***	−3.709 ***	−1.199 ***
	(0.282)	(0.208)	(0.828)	(0.457)
Higher education	−0.746	−0.813 *	−1.050	−1.437
	(0.621)	(0.430)	(1.827)	(0.944)
Marital status	−0.421 ***	−0.644 ***	0.456	0.611 *
	(0.125)	(0.161)	(0.368)	(0.353)
Political status	−0.124	−0.106	0.171	−0.978 ***
	(0.295)	(0.159)	(0.869)	(0.349)
Religious belief	−0.252	−0.0926	−1.032 **	−0.249
	(.0155)	(0.238)	(0.457)	(0.524)
Household living expenditure (ln)	0.110 ***	0.114 ***	−0.0488	0.0813
	(0.025)	(0.029)	(0.072)	(0.638)
Smoking status	−0.179	−0.415 ***	1.195 *	0.611 **
	(0.235)	(0.125)	(0.689)	(0.275)
Drinking status	−0.535 ***	−0.740 ***	0.0089	−0.968 ***
	(0.167)	(0.124)	(0.490)	(0.273)
Residential location	−0.501 ***	−0.225	−2.019 ***	−1.394 ***
	(0.138)	(0.153)	(0.408)	(0.336)
Health insurance	−0.0968	−0.770 *	−2.360 ***	0.116
	(0.288)	(0.418)	(0.848)	(0.918)
Constant	−1.360 *	0.0383	42.24 ***	26.78 ***
	0.718)	(0.862)	(2.113)	(1.891)
Observations	3117	3206	3117	3206
*R* ^2^	0.104	0.093	0.080	0.049
Adjusted *R*^2^	0.100	0.089	0.076	0.045

Note: Standard errors in brackets, * *p* < 0.1, ** *p* < 0.05, *** *p* < 0.01; ^a^ means primary school and below.

**Table 4:** Regression Results by Residential Location.

Variable	Model 4-1		Model 4-2	
	ADL (Physical Health)		CES-D (Mental Health)	
	Rural Area	Urban Area	Rural Area	Urban Area
Internet use	−0.805 **	−0.631 ***	−4.011 ***	−0.822
	(0.317)	(0.226)	(0.834)	(0.534)
Age	0.0905 ***	0.116 ***	−0.231 ***	−0.122 ***
	(0.007)	(0.011)	(0.019)	(0.028)
Gender	−0.816 ***	−0.704 ***	−2.029 ***	−0.857 *
	(0.124)	(0.204)	(0.325)	(0.481)
Education level ^a^				
Junior high school	−0.543 ***	−0.372 *	−1.292 ***	−0.833 *
	(0.153)	(0.207)	(0.403)	(0.489)
Senior high school	−0.939 ***	−0.329	−2.676 ***	−1.384 **
	(0.236)	(0.235)	(0.621)	(0.556)
Higher education	−0.249	−0.854 **	0.660	−1.509
	(−0.859)	(0.394)	(2.256)	(0.932)
Marital status	−0.511 ***	−0.578 ***	0.641 **	0.596
	(0.115)	(0.194)	(0.303)	(0.460)
Political status	−0.129	−0.140	−0.181	−1.332 **
	(0.175)	(0.219)	(0.461)	(0.518)
Religious belief	−0.102	−0.420	−0.859 **	−0.551
	(0.154)	(0.261)	(0.405)	(0.617)
Household living expenditure (ln)	0.113 ***	0.103 ***	−0.0194	0.0947
	(0.021)	(0.038)	(0.057)	(0.092)
Smoking status	−0.419 ***	−0.179	0.506	0.694
	(0.123)	(0.215)	(0.324)	(0.510)
Drinking status	−0.609 ***	−0.852 ***	−0.735 **	−0.777 *
	(0.115)	(0.188)	(0.302)	(0.445)
Health insurance	−0.523 **	0.749	−1.121 *	−3.858 **
	(0.258)	(0.684)	(0.678)	(1.619)
Constant	0.265	−3.100 ***	38.09 ***	29.44 ***
	(0.631)	(1.181)	(1.657)	(2.794)
Observations	4612	1711	4612	1711
*R* ^2^	0.127	0.156	0.064	0.044
Adjusted *R*^2^	0.125	0.150	0.061	0.036

Note: Standard errors in brackets, * *p* < 0.1, ** *p* < 0.05, *** *p* < 0.01; ^a^ means primary school and below.

**Table 5:** Regression Results by Age.

Variable	Model 5-1		Model 5-2	
	ADL (Physical Health)		CES-D (Mental Health)	
	Age = 60–69	Age ≥ 70	Age = 60–69	Age ≥ 70
Internet use	−0.727 ***	−0.926 **	−1.353 ***	−1.637
	(0.204)	(0.380)	(0.483)	(1.041)
Gender	−0.718 ***	−0.629 ***	−1.744 ***	−2.469 ***
	(0.138)	(0.169)	(0.323)	(0.463)
Education level ^a^				
Junior high school	−0.422 ***	−1.160 ***	−1.234 ***	0.0882
	(0.144)	(0.227)	(0.341)	(0.623)
Senior high school	−0.839 ***	−0.760 **	−1.990 ***	−0.920
	(0.194)	(0.298)	(0.461)	(0.817)
Higher education	−1.049 *	−0.551	−2.013	−1.041
	(0.539)	(0.478)	(1.275)	(1.311)
Marital status	−0.352 ***	−1.179 ***	0.176	2.489 ***
	(0.132)	(0.148)	(0.313)	(0.407)
Political status	−0.152	0.113	−1.529 ***	−0.487
	(0.183)	(0.208)	(0.435)	(0.570)
Religious belief	−0.231	−0.179	−1.014 **	−0.596
	(0.177)	(0.203)	(0.421)	(0.558)
Household living expenditure (ln)	0.123 ***	0.0901 ***	0.0955	−0.0609
	(0.025)	(0.029)	(0.061)	(0.078)
Smoking status	−0.293 **	−0.625 ***	0.630 *	1.003 **
	(0.135)	(0.175)	(0.321)	(0.481)
Drinking status	−0.775 ***	−0.646 ***	−1.060 ***	0.00382
	(0.124)	(0.161)	(0.294)	(0.441)
Residential location	−0.356 ***	−0.230	−2.228 ***	−1.633 ***
	(0.131)	(0.166)	(0.310)	(0.457)
Health insurance	−0.277	−0.584 *	−0.520	−1.700 *
	(0.367)	(0.332)	(0.868)	(0.912)
Constant	5.657 ***	7.731 ***	21.52 ***	20.51 ***
	(0.408)	(0.388)	(0.965)	(1.063)
Observations	3551	2772	3551	2772
*R* ^2^	0.084	0.100	0.077	0.033
Adjusted *R*^2^	0.080	0.096	0.074	0.028

Note: Standard errors in brackets, * *p* < 0.1, ** *p* < 0.05, *** *p* < 0.01; ^a^ means primary school and below.

**Table 6:** The Treatment of Endogenity: Instrumental Variable Model.

	Model 6-1	Model 6-2
	ADL Score (Physical Health)	CES-D Score (Mental Health)
Internet use	−1.938 ***	−5.335 ***
	(0.516)	(1.324)
Control variables	Y	Y
Constant	−0.143	36.39 ***
	(0.554)	(1.421)
Observations	6323	6323
Adjusted *R*^2^	0.130	0.063
F statistics of the first stage regression	896.91	896.91
DWH test	*p* = 0.0096	*p* = 0.0053

Note: Standard errors in brackets, *** *p* < 0.01.

**Table 7:** The Mediating Effect of Social Capital.

Variable	Model 7-1	Model 7-2	Model 7-3	Model 7-4	Model 7-5	Model 7-6
	ADL	Social Capital	ADL	CES-D	Social Capital	CES-D
Internet use	−0.690 ***	0.363 ***	−0.628 ***	−1.891 ***	0.363 ***	−1.834 ***
	(0.182)	(0.547)	(0.182)	(0.465)	(0.055)	(0.467)
Control variables	Y	Y	Y	Y	Y	Y
Social capital			−0.170 ***			−0.156
			(0.042)			(0.107)
Constant	−0.365	0.385 *	−0.300	35.77 ***	0.385 *	35.83 ***
	(0.546)	(0.164)	(0.546)	(1.399)	(0.164)	(1.400)
Observations	6323	6323	6323	6323	6323	6323
*R* ^2^	0.137	0.036	0.139	0.071	0.036	0.071
Adjusted *R*^2^	0.135	0.034	0.1369	0.069	0.034	0.069

Note: Standard errors in brackets, * *p* < 0.1, *** *p* < 0.01.

## Data Availability

The data of CHARLS2018 is publicly available at http://charls.pku.edu.cn/ accessed on 31 July 2019.
